# The Molecular Architecture of Somatic Spines of the Lateral Septum

**DOI:** 10.1002/cne.70195

**Published:** 2026-08-29

**Authors:** Daniela Hacker, Erich Weisheim, Michaela Schweizer, Undine Schneeweiß, Maximilian Borgmeyer, Michael Brecht, Marina Mikhaylova

**Affiliations:** ^1^ AG Optobiology, Institute of Biology Humboldt Universität zu Berlin Berlin Germany; ^2^ Morphology and Electron Microscopy, Center for Molecular Neurobiology (ZMNH) University Medical Center Hamburg‐Eppendorf Hamburg Germany; ^3^ Bernstein Center for Computational Neuroscience Berlin Humboldt‐Universität zu Berlin Berlin Germany; ^4^ Institute for Translational Medicine MSH Medical School Hamburg Hamburg Germany; ^5^ NeuroCure Cluster of Excellence Humboldt‐Universität zu Berlin Berlin Germany

**Keywords:** lateral septum, organelles, somatic spines

## Abstract

The lateral septum is a key subcortical structure and has been implicated in social memory. One aspect of social memory, the ability to recognize relatives, is conserved across vertebrate species and reflected in stable, lifelong memories. Synapses are considered to be the smallest unit of memory storage. Excitatory synapses are typically found on dendritic spines, whereas inhibitory synapses are mostly located on the dendritic shaft. Here, we investigate the synaptic architecture of unusual somatic spines, an apparent synaptic specialization of septal GABAergic neurons. We uncover the formation and molecular organization of septal somatic spines found in the lateral septum in in vivo and in vitro model systems using various microscopy approaches. We use classical label‐free methods such as transmission electron microscopy and Golgi stainings and established new culturing methods for dissociated and organotypic septal slices that were kept in culture over multiple weeks. We describe the presence, morphology, ultrastructure, and molecular composition of excitatory somatic spines across multiple developmental stages in various model systems. We propose that the ability to develop such spines is an intrinsic feature of somatospiny neurons that does not depend on extra‐septal connectivity. While smaller than dendritic spines, somatic spines exhibited distinct features, frequently containing secretory organelles such as autophagosomes, multivesicular bodies, and endosomes, but often lacking a spine apparatus and ribosomes. Our findings offer insights into the molecular architecture of septal somatic spines and establish a basis for further investigations into the somatic spines of the lateral septum.

## Graphical abstract

Spine synapses represent stable synaptic connections that allow neurons to chemically communicate with each other. Although known for decades, the unusual spine synapses found directly on neuronal cell bodies in the lateral septum have not been characterised in detail. Using traditional, unbiased approaches such as electron microscopy and Golgi stainings, we replicate and extend the initial findings. Additionally, using fluorescent microscopy of dissociated and organotypic septal cultures, we characterise the development and composition of septal somatic spines. We find that they are excitatory, contain membranous organelles and develop independently of extra‐septal input. We thus describe model systems suitable for the investigation of somatospiny neurons of the lateral septum.

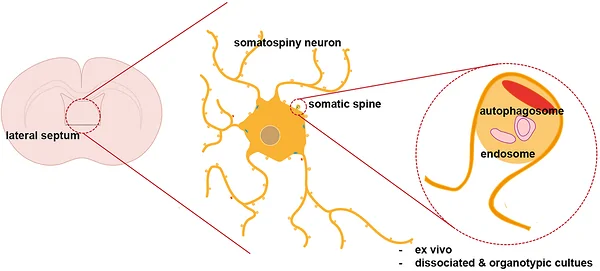

## 1 Introduction

The brain is made up of various neuronal cell types and many different synapse subtypes. Differences in their properties include the type of signal transmission, synaptic vesicle (SV) count, synapse strength, size, shape, localization, and molecular composition. The introduction of cell type‐specific Fluorescence Activated Synaptosome Sorting (FASS) allowed the cell type‐specific analysis of synapse subtypes (Biesemann et al. 2014; Kaulich et al. 2024; van Oostrum et al. 2023). While this technique allows for a high‐throughput analysis of synapses of a specific cell type, by itself, it does not grant information on their subcellular localization. Dendritic spine morphology plays a crucial part in plasticity and stability of the synapse and can be broadly categorized into mushroom, stubby, thin, and filopodia‐like, ranging from mature to immature spines (Pchitskaya and Bezprozvanny 2020).

An unusual neuronal subtype distinguished by its morphology is the somatospiny neuron, characterized by the presence of dendritic spines on the soma. In the lateral septum, a substantial proportion of spiny neurons has been described (Jakab and Leranth 1990b; Onteniente et al. 1987). These somatospiny neurons have been proposed to be GABAergic (Jakab and Leranth 1990a; Onteniente et al. 1987). However, because GABAergic neurons constitute the vast majority of neurons in the lateral septum (75%–100% of neurons) (Risold and Swanson 1997; Rizzi‐Wise and Wang 2021; Wong et al. 2016; Zhao et al. 2013), this classification does little to distinguish somatospiny neurons from other septal neurons, and to date, no systematic description of their synaptome exists. Observations regarding the identity of somatospiny cells and the cells innervating them have been made (Jakab et al. 1991; Jakab and Leranth 1990a, 1990b, 1991). Their somatic spines have further been suggested to receive excitatory hippocampal, vasopressinergic, and catecholaminergic inputs (Jakab et al. 1991; Jakab and Leranth 1990a, 1990b, 1991, 1993). However, to date, no systematic description of their synaptome exists.

The exact region of the lateral septum in which somatospiny neurons are found harbors a population of neurons mediating kinship memory (Clemens et al. 2020). Here, the connectivity between the septum and hippocampus mediates whether or not rats prefer the presence of their relatives (Clemens et al. 2020). While younger animals prefer the company of littermates, older animals prefer the presence of nonrelated pups. Lesioning the lateral septum abolishes these preferences (Clemens and Brecht 2021).

Generally, excitatory glutamatergic synapses on dendritic spines used to be considered more stable and mature than, for example, dendritic shaft synapses (Fanutza et al. 2025; Grutzendler et al. 2002). Excitatory synapses are predominantly formed on dendritic spines, but can also occur on the dendritic shaft, with their relative distribution depending on both neuronal maturity and cell type (Bucher et al., 2020; van Bommel et al., 2019); the abundance of each depends not just on maturity but also on the specific cell types. Hippocampal CA1 parvalbumin neurons, for example, are known to receive most of their excitatory input on dendritic shaft synapses (Foggetti et al. 2019).

A structure often found in stable and mature dendritic spines is the spine apparatus (Konietzny et al. 2022, 2023; Yap et al. 2020), a specialized endoplasmic reticulum‐derived organelle (Spacek and Harris 1997). Spines can also contain other organelles such as ribosomes and recycling endosomes, which can provide the synapse with membrane and proteins to be used, for example, during plasticity‐induced remodeling (Park et al. 2006). Although abundantly present in the dendritic shaft (Spacek and Harris 1997), endosomes and other organelles were seen in less than 20% of spines in the hippocampus (Cooney et al. 2002) despite them being required for the enlargement of spines (Park et al. 2006). In addition to glutamatergic synapses, GABAergic inhibitory inputs can be found along dendrites as well as on the soma and the axon initial segment (Kwon et al. 2019; Lipkin and Bender 2023; Vereczki et al. 2016).

Despite the considerable abundance of somatospiny neurons in the lateral septum, their biology remains largely unexplored, mainly due to the lack of well‐established model systems. Thus, the developmental origins and subcellular and molecular composition of septal somatic spines are still unknown. Here, we present novel methods to investigate these structures and aim to elucidate the development and molecular characteristics of somatospiny neurons within a predominantly GABAergic environment.

## 2 Materials and Methods

### 2.1 Antibodies

The antibodies used in this study are listed below.

Primary antibodies:

**Table:** 

Name	Usage and dilution	Species	Supplier	Identifier
GFP	IHC: 1:250	Camelid	NanoTag	N0304‐At488‐L
Homer1/2/3	IHC: 1:500	Rabbit	Synaptic Systems	160103
PSD95	IHC: 1:500	Mouse	neuromab	75‐028
PSD95	IHC: 1:500	Camelid	NanoTag	N3702‐SC3‐L
Shank3	IHC: 1:500	Rabbit	Synaptic Systems	162302
Shank3	IHC: 1:500	Guinea pig	Synaptic Systems	162304
Synaptopodin	IHC: 1:200	Guinea pig	Synaptic Systems	163004
Synaptopodin	IHC: 1:250	Rabbit	Synaptic Systems	163002
Synaptopodin	IHC: 1:500	Mouse	Origene	BM5086P
Gephyrin	IHC: 1:200	Guinea pig	Synaptic Systems	147318
Gephyrin	IHC: 1:1000	Mouse	Synaptic Systems	147011
Bassoon	IHC: 1:500	Mouse	Enzo	ADI‐VAMPS003‐F13
Bassoon	ICC: 1:500	Guinea pig	Synaptic Systems	141004
Bassoon	ICC: 1:500	Rabbit	Synaptic Systems	141013
MAP2‐488	ICC: 1:500	Mouse	Merck Millipore	MAB3418X
MAP2	ICC: 1:1000	Rabbit	Synaptic Systems	188002
NeuN‐488	ICC 1:500	Mouse	Merck Millipore	MAB377X
Calbindin D‐28K	ICC 1:500	Mouse	Sigma‐Aldrich	C9848
GAD65	ICC 1:500	Rabbit	proteintech	21760‐1‐AP

Secondary antibodies:

**Table:** 

Antibody	Conjugated fluorophore	Usage and dilution	Supplier	Identifier
Anti‐mouse	647	IHC: 1:500	Life Technologies	A‐21236
Anti‐rabbit	568	IHC: 1:500	Life Technologies	A‐11036
Anti‐guinea pig	568	IHC: 1:500	Life Technologies	A‐11075
Anti‐guinea pig	635P	IHC: 1:500	Abberior	ST635P‐1006‐500UG
Anti‐guinea pig	Alexa Fluor 405	IHC: 1:500	Abcam	ab175678
Anti‐mouse	Alexa647	IHC: 1:500	Southern Biotech	1144‐31
Anti‐rabbit	Alexa488	IHC: 1:500	Life Technologies	A‐11034
Anti‐rabbit	Alexa405	IHC: 1:500	Abcam	ab175652
Anti‐mouse	Alexa647	IHC: 1:500	Life Technologies	A‐21236
Anti‐rabbit	Abberior STAR 635P	IHC: 1:500	Abberior	2‐0012‐007‐2
Phalloidin	Atto 647N	IHC: 1:100	Sigma	65906‐10NMOL
Anti‐mouse	STAR580	ICC: 1:500	Abberior	2‐0002‐005‐1
Anti‐rabbit	Alexa647	ICC: 1:500	Life Technologies	A‐21245
Anti‐mouse	Alexa568	ICC: 1:500	Life Technologies	A‐11031

### 2.2 Animals

Experimental procedures using Wistar rats (RjHan:WI, RRID:RGD_13508588) were authorized under T‐HU‐03‐23, G 0095/21, and G 0149/23 under the local authority LaGeSo Berlin in accordance with the European Communities Council Directive (2010/63/EU) and the Animal Welfare Law of the Federal Republic of Germany (Tierschutzgesetz der Bundesrepublik Deutschland, TierSchG). For all dissociated and organotypic culture preparations, animals were not differentiated according to their sex.

### 2.3 Dissociated Septal Neuron Cultures From Postnatal Day 0 (P0)/P1 Rats

P0/P1 Wistar (RjHan:WI) rats were decapitated, and the brain and the septi were isolated from 1‐mm‐thick coronal slices obtained from a tissue chopper. All tissue isolation steps were done in ice‐cold HBSS. Once septa from all animals were collected in 10–15 mL HBSS, they were carefully washed five times with 9 mL of ice‐cold HBSS under a flow hood. For each septum, 100 µL of 0.25% Trypsin/EDTA solution was added to the septa, which were suspended in 2 mL of HBSS. After 15 min at 37°C, Trypsin was removed by washing five times with 9 mL of 37°C‐warm HBSS. The remaining 2 mL of HBSS–tissue mixture was moved to a 2‐mL reaction tube and triturated with 20G/26G needles. The cell solution was filtered (Easy Strainer, Greiner 542000), and the filter was washed with HBSS. Cells were then manually counted in a Neubauer chamber and plated at densities between 40,000 and 80,000 cells/mL in prewarmed full medium (Dulbecco's modified Eagle's medium [DMEM], 10% fetal bovine serum [FBS], 1× penicillin/streptomycin, 2 mM glutamine). After 1 h at 37°C and 5% CO_2_, the medium was exchanged for 37°C‐warm Brain Phys medium (Gibco) with added 1× SM1 and 0.5 mM glutamine supplements. To counteract a change in osmolarity due to evaporation of the medium, after 1 week, 10% of the medium was removed, and 20% fresh medium was added.

### 2.4 Organotypic Slices Culture

Organotypic slice culture preparation was adapted from Gee et al. (2017). Briefly, P4–7 Wistar (RiHan:WI) rats were decapitated, and the brain was isolated, placed onto sterile filter paper, and slightly rinsed with dissection medium (248 mM sucrose, 26 mM NaHCO_3_, 10 mM D‐glucose, 4 mM KCl, 5 mM MgCl_2_, 1 mM CaCl_2_, 0.001% phenol red, Milli‐Q water was added to 500 mL; osmolality should be 310–320 mOsm kg and pH >8 [red‐magenta]; before use, 1 mL kynurenic acid was added to 50 mL, and the solution was bubbled with O_2_ until orange). Afterward, the brain was sliced into either 400‐ or 600‐µm‐thick coronal slices using a McIlwain TC752 tissue chopper in the anterior–posterior direction and carefully transferred into ice‐cold dissection medium. Slices containing the septum were isolated, and the septum was isolated from the slices with two cuts using tweezers. Septal slices were then transferred under a flow hood and gently placed onto membranes that were sitting in slice culture medium (394 mL MEM, 100 mL heat‐inactivated horse serum, 1 mM L‐glutamine, 0.01 mg/mL insulin, 14.5 mM NaCl, 2 mM MgSO_4_, 1.44 mM CaCl_2_, 0.00125% ascorbic acid, 13 mM D‐glucose) at 37°C and 5% CO_2_. After placement, slices were moved to incubators at 37°C and 5% CO_2_ before new slices were isolated. Cultures were fed twice a week every 2–3 days by removing the old slice culture medium and adding 37°C‐warm new medium.

### 2.5 Cell Electroporation in Organotypic Slices

Organotypic slices were electroporated according to Kawabata et al. (2004). NEPA21 electroporator was equipped with CUY700P3E and CUY700P3L electrodes. The slice was positioned on top of the electrode on its membrane. The cathode was filled with slice EP buffer. After positioning the slice and the membrane, a drop of 0.5 µg/µL DNA diluted in slice EP buffer was pipetted on top. The anode was placed on the liquid's surface, and a poring pulse was initiated, followed by a transfer pulse as specified in the manufacturer's protocols. The poring pulse was set to 50 V with a pulse length of 5 ms and a decay rate of 10%. The transfer pulse was set to 20 V with a pulse length of 50 ms and a decay rate of 40%. Electroporation was performed using AC.

### 2.6 Microscopy—Preparation

#### 2.6.1 Immunocytochemistry (ICC) of Dissociated Cultures

If not described otherwise, neurons at various days in vitro (DIVs) were fixed by replacing the culture medium with 4% paraformaldehyde (PFA)/4% sucrose for 10 min. The fixative was then washed off three times for 10 min with PBS. To permeabilize the cells, they were incubated with PBS containing 0.25% Triton X‐100, which was removed with two 5‐min wash steps with PBS. Coverslips were blocked with blocking buffer (PBS containing 10% heat‐inactivated horse serum [30 min at 65°C] and 0.1% Triton X‐100; BB‐HE). Coverslips were then incubated overnight at 4°C with primary antibody diluted in BB‐HE. If used, the cells were incubated with Phalloidin at the same time. The next day, the primary antibody was washed off with three 10‐min PBS washing steps. Suitable secondary antibodies were diluted in blocking buffer and incubated for 1 h at room temperature. The secondary antibody was washed off three times for 10 min with PBS. When necessary, prelabeled primary antibodies were diluted in blocking buffer and added to the cells at this stage and incubated at 4°C overnight. The antibody was then washed off with three 10‐min PBS wash steps. Coverslips were then mounted on glass coverslips with the cells facing a Mowiol droplet on the glass coverslip. Samples could be imaged ∼3 h after air‐drying them in the dark.

#### 2.6.2 Immunohistochemistry (IHC) of Cryosections

Cryosections obtained from perfused rat/mouse brains were cut with a cryostat to 30–40 µm thickness. For this procedure, the perfused brains were incubated with 30% sucrose in PBS to prevent ice‐crystal formation and then frozen at −80°C and cut at −20°C coronally with a cryostat. Blade and stage temperature were adjusted to around −20°C maximum temperature −15°C minimum temperature−25°C. Sections were incubated with primary antibody diluted in blocking buffer BB‐HE on a horizontal shaker at 4°C for at least 36 h. Sections were washed with PBS twice for a total of 1 h, followed by two to three wash steps with PBS containing 0.2% BSA for 1 h. Secondary antibody was diluted in blocking buffer and incubated with the sections for 2 h at room temperature. Sections were then washed three times with PBS. If desired, DAPI was added at 1:500 in the second‐to‐last wash step. For mounting, slices were rinsed in tap water and gently placed on glass slides that were air‐dried. A drop of Mowiol was placed on the slice, and a coverslip was fixed on the sample.

#### 2.6.3 IHC of Organotypic Slices

PFA‐fixed organotypic slices as described above were permeabilized with 0.4% Triton X‐100 in PBS for 30–90 min. After three washes with PBS, the slices were quenched using 50 mM NH_4_Cl followed by three washes with PBS and blocking for 1 h. The primary antibody was diluted in blocking buffer, and slices were incubated for 72 h at 4°C. After three washes with PBS, secondary antibody was diluted in blocking buffer and incubated with the slices overnight. Slices were then washed three times with PBS and optionally incubated with DAPI 1:500, before another PBS wash step. Slices were mounted using Mowiol.

#### 2.6.4 Golgi Staining

Brains were stained according to FD Rapid GolgiStain Kit—FD NeuroTechnologies. P7 and P14 brains were briefly incubated in 0.5% PFA directly following the dissection. In brief, P7, P14, and adult brains were incubated in impregnation solution for about 2 weeks. During this incubation time, the impregnation solution was exchanged once. Afterward, the brains were washed twice with protection solution for up to 1 week. To prepare the brains for freezing, they were incubated in cryoprotection solution (30% sucrose in PBS). They were then placed at −80°C and stored until they were sectioned at a thickness of 200 µm with a cryostat. They were mounted on gelatin‐coated slides using protection solution. Slides were thoroughly washed with tap water for 1 h and additionally for 10 min with dH_2_O. They were coated by incubating for 1 min with a 1% gelatine, 0.1% chromalaun solution. They were stored vertically at room temperature, protected from dust until further use.

After air‐drying the mounted slices, they were stained on the slide in a glass container. They were rinsed with double‐distilled water twice and then incubated in staining solution for 10 min. Afterward, they were sequentially dehydrated for 4 min/solution by adding ethanol in ascending concentrations, starting with 50% ethanol, 70% ethanol, and 95% ethanol. Dehydration with 100% ethanol was performed four times for 4 min each. Sections were then cleared with xylene three times for 4 min and covered with a glass coverslip.

#### 2.6.5 TEM Preparation

Rats were deeply anesthetized and sacrificed by transaortic perfusion with isotonic saline followed by a mixture of fixatives containing 4% PFA and 1% glutaraldehyde in 0.1 M phosphate buffer (PB), pH 7.3. Brains were removed and postfixed overnight in the same fixative. Frontal sections (100 µm) cut on a vibratome (Leica VT 1000S) were rinsed in several changes of PB, and sections containing septal areas were osmicated in 1% osmium tetroxide in 0.1 M cacodylate buffer for 20 min. These sections were further dehydrated using ascending ethyl alcohol concentration steps, followed by two rinses in propylene oxide. Infiltration of the embedding medium was performed by immersing the samples in a 1:1 mixture of propylene oxide and epon and finally in neat epon, and the samples were then polymerized at 60°C. Semithin sections (0.5 µm) were prepared for light microscopy, mounted on glass slides, and stained with 1% toluidine blue. Areas of interest were closely trimmed.

### 2.7 Microscopy

#### 2.7.1 Spinning Disk Confocal Imaging

Fixed confocal imaging was performed using a custom‐built inverted microscope equipped with an Olympus IX83 body. For widefield, an LED lamp was used. Excitation lasers used were 405, 488, 561, and 640 nm. Lasers were coupled to a CSU‐W1 spinning disk unit. Corresponding filters were used, and two cameras (sCMOS pco.edge and Andor EMCCD) were used for acquisition. Fixed imaging was performed using a 60x (Olympus, UPLXAPO60XO, 1.42 NA) objective.

#### 2.7.2 Scanning Confocal Imaging

Additional fixed confocal imaging to determine the number of septal neurons in our cultures was performed using the commercially available Leica SP8 scanning confocal microscope. Excitation lasers used were 405, 488, 594, and 633 nm. A HyD detector was used to collect the emitted light, and the pinhole was set to 1.0 Airy unit. Pixel size was set to 542 nm and scan speed to 400 Hz. Volumetric scans were performed with 1 µm between *z*‐positions. An HC PL APO CS2 40x/1.30 oil objective was used for TileScan images that were merged using the LasX software. An overlap of 15% was set.

#### 2.7.3 Widefield Imaging for Golgi Stainings

Golgi overview images were obtained from a Nikon Ni‐E system. Widefield illumination was used for excitation, and a Nikon DS‐Ri2 color camera was used to collect light at a resolution of 4908 × 3264 pixels, resulting in a pixel size of 365 nm/pixel. A Nikon Plan Apo λ 20x NA 0.75 objective was used. An overlap of 15% was set for overview images. For ages P7, P14, and adult rat, sections from *N* = 2, 3, and 2 animals were imaged, respectively.

Representative close‐up images of somata and their spines were imaged using an Olympus cellSens setup. Differential interference contrast (DIC) was used, and a Hamamatsu Orca R2 camera with a pixel size of 6.45 µm was used. A UPLSAPO 60x/1.35 Oil objective was used. Volumetric scans were performed with 0.31 µm between *z*‐positions.

Representative images of dendrites and their spines were imaged using an Olympus Bx53 setup with an Olympus DP28 camera and the Olympus UplanX Apo Oil 100x objective.

#### 2.7.4 TEM

Images were acquired with a JEM‐2100Plus Transmission Electron Microscope at 200 kV (Jeol) equipped with a XAROSA CMOS camera (EMSIS).

#### 2.7.5 Image Analysis

##### 2.7.5.1 TEM Analysis

Spine length, spine area, PSD width, spine head width, spine base width, membrane length, and presynaptic SV count were measured as indicated in Figure 2E in 2D images, as well as spine morphology. SVs were counted manually. Circular, small vesicles of around 40 nm in a presynaptic site opposing a PSD were considered SVs. Several somatic (*n* = 16) and dendritic (*n* = 19) spines from two adult rats *N* = 2 were analyzed. When, for example, the outlines of structures were unclear, the spine was excluded from the analysis. Since changes in spine morphology are gradual, the classifications often differ from one person to the next. For that reason, the analysis was done by two independent scientists and compared afterward. In cases when the classification differed, the expert's classification was used. Spine morphology was classified as stubby (spines without elongation, or a discernible spine neck), long (elongated spines that do not have a significantly wider spine head than the spine base width), and mushroom (spines that have a thin spine neck followed by a spine head wider than the spine base). Spines were classified as excitatory when the postsynaptic density was by eye clearly more electron dense than the presynaptic site (*n* = 14 somatic and *n* = 22 dendritic spines, *N* = 2 animals). To determine the spine area that was occupied by organelles, we only considered spines that contained organelles. The organelles were classified according to their morphology.

##### 2.7.5.2 Dissociated Culture Analysis

To determine the number of somatic spines at different DIVs without the need for overexpression, maximum projections stained for the somatodendritic marker MAP2, the presynaptic marker bassoon, and the excitatory scaffold SHANK3 were used for *n* = 65 cells (*N* = 9 independent preparations). An overlap of pre‐ and postsynaptic puncta <10 µm from the soma was counted as a somatic spine. Low culture density allowed the confident identification of somatic spines. Coverslips were imaged at a spinning disk confocal microscope (see above).

To determine the percentage of septal neurons in our dissociated cultures, we co‐stained DIV7–8 cultures with the neuronal marker NeuN, the GABAergic neuron marker GAD65, and calbindin, which is enriched in GABAergic neurons of the septal area (Zhao et al. 2013). Coverslips were imaged on a scanning confocal microscope. We imaged large tile scans to image a sufficient number of cells. Tile scans were processed with FIJI, and using the plugin StarDist2D (Weigert et al. 2020), we generated ROIs of NeuN+ neurons in maximum projected images with a Gaussian filter of 1.0. It was then manually determined whether cells were positive for calbindin or GAD65.

##### 2.7.5.3 Organotypic Slice Culture Analysis

In septal organotypic cultures, maximum projections of cells electroporated with a mRuby2 or GFP cell fill were used to determine the number of somatic spines at different DIV of *n* = 187 cells (*N* = 15 animals). Sparse labeling with field electroporation allowed the confident identification of somatic spines and allowed the visualization of spine heads and spine necks.

To determine the molecular composition of somatic spines, slices electroporated and imaged as described previously were additionally stained against one to two synaptic markers per group. The presence of markers in somatic spines (as determined described above) was confirmed manually for somatic and dendritic septal spines.

### 2.8 Statistics

Before parametric statistical analysis, we checked for outliers using the ROUT test with *Q* = 1%. We then used the D'Agostino and Pearson test to assess the normal distribution of our datasets. When comparing two groups, two‐tailed *t*‐tests or Mann–Whitney *U* tests were used when data were or were not normally distributed, respectively. GraphPad Prism was used for all statistical analyses.

## 3 Results

### 3.1 Somatospiny Neurons Could Be Identified in the Lateral Septum Through Golgi Staining

**FIGURE 1:**
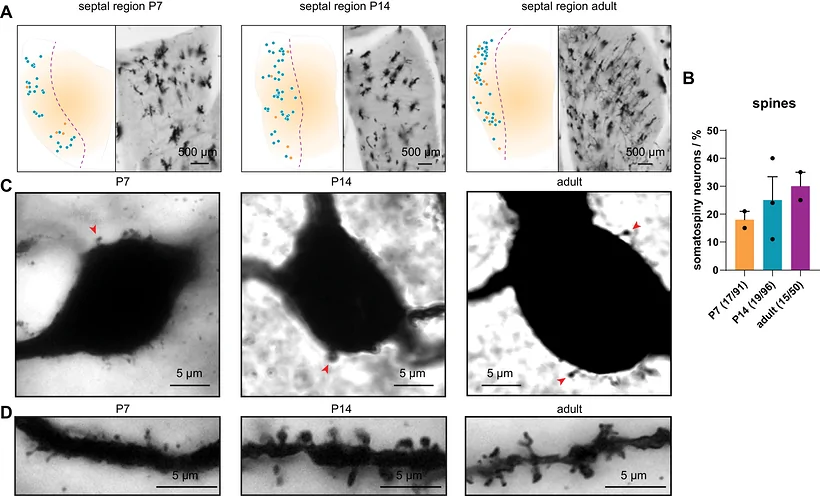
Somatospiny neurons in the rat lateral septum identified by Golgi staining. (A) Septal area of 200‐µm‐thick slices from Golgi‐stained P7, P14, or adult rat brains, with only one z‐plane shown. Scale bar, 250 µm. Representative, rough maps of the lateral septum in the left half of each age. Neurons that were considered somatospiny (orange dots) and not somatospiny (blue dots) are marked. (B) Share of somatospiny neurons in *z*‐projections of the lateral septum in slices as shown in A. In brackets: number of somatospiny neurons/total number of neurons counted. (C, D) Representative images of somatospiny neurons found in the lateral septum, one example per age (C) and one of their dendrites (D). Arrows indicate somatic spines. Scale bars are indicated in the images. *N* = 2 (P7); 3 (P14); and 2 (adult) animals.

Our initial goal was to visualize the somatic spines first described in septal GABAergic neurons (Onteniente et al. 1987) by reproducing the original findings of Jakab and Leranth (1990a, 1990b) using similar methods. To analyze large areas of the septum without relying on genetic manipulation, we employed Golgi–Cox staining. Structures were identified as somatic or dendritic spines when their neck was clearly connected to the soma or dendrite of a neuron, respectively. Here, we could reveal the morphology of somatospiny septal neurons and confirmed their widespread distribution throughout the lateral septum of adult rats (Figure 1A–D). Interestingly, we found that these spines were not just present in adult rat brains, but in brains from as early as P7 and P14. In the P7 rat septum, 18% of lateral septal neurons were somatospiny. This increased to 25% in P14 and up to 30% in the adult brain. This was comparable to the initial findings that 20% of lateral septal neurons in adult Sprague–Dawley rats are somatospiny (Jakab and Leranth 1990). We classified spines based on their morphology, with discernible spine head and neck, making them unlikely to be nonsynaptic protrusions such as filopodia, but rather spine synapses.

### 3.2 Somatic Spines Are Frequently Stubby and Smaller Than Dendritic Spines

**FIGURE 2:**
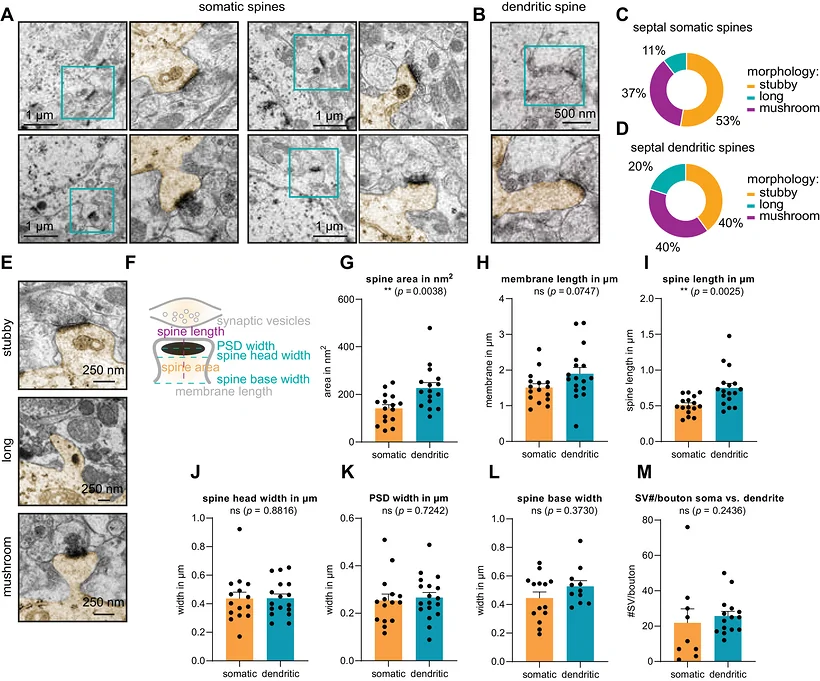
Somatic spines are frequently stubby and smaller than dendritic spines. (A, B) Representative transmission electron microscopy images of septal somatic (A) and dendritic (B) spines of adult rats (*N* = 2); scale bar, 1 µm (A) and 500 nm (B). (C, D) Spine morphology characterization of images as shown in (A) and (B), respectively. Morphology was grouped into stubby, long filamentous and mushroom spines (*n* = 19 somatic spines and *n* = 25 dendritic spines, *N* = 2). Scale bars according to each image. (E) Example images of stubby, long, and mushroom spines. (F) Schematic depicting spine characterization measurements depicted in (F)–(L). (G–M) Quantification of spine area (G), membrane outlining spines (H), spine length (I), spine head width (J), PSD width (K), width at the base of spines (L), and number of SVs on the opposing presynaptic site (M) compared between somatic (*n* = 19) and dendritic (*n* = 26) spines (shown: cell averages of *N* = 2 animals). Spines were excluded from individual measurements when the measurement would be imprecise due to, for example, low contrast. Normality was determined according to the D'Agostino and Pearson test; the appropriate unpaired *t*‐test was selected accordingly. **, *p* = 0.0038 (G); ns, *p* = 0.0747 (H); *, *p* = 0.0025 (I); ns, *p* = 0.8816 (J); ns, *p* = 0.7242 (K); ns, *p* = 0.3730 (L); *, *p* = 0.2436 (M).

While Golgi staining allows for an unbiased approach to observe the morphology of multiple cell types, it comes short in the characterization of molecular features of subcellular structures. To address the question of how the ultrastructure of somatic spines is organized, we moved on to high‐resolution transmission electron microscopy (TEM). TEM allows for the visualization of membranes and protein densities and allowed us to conclusively confirm that the spine‐like structures emerging from neuronal somata contain a profound postsynaptic density (PSD), a defining feature of excitatory synapses. It further allowed us to confirm that they are opposed by presynaptic sites containing presynaptic vesicles and are not merely immature filopodia. Thus, we could confirm the presence of somatic spines similar to Jakab and Leranth (1990b) (Figure 2A).

Spine synapses are typically found on dendrites, so their presence on the neuronal soma could have important implications for how the synapses influence the cell's output. To determine whether the subcellular localization also provided somatic spines with unique properties, distinguishing them from dendritic spines, we compared our findings with the characteristics of dendritic spines in the lateral septum (Figure 2B). Since spine morphology can point to their developmental stage and indirectly to their function, we first classified them. We observed that most septal somatic spine synapses were stubby, which was defined as not containing a discernible spine neck, but their spine head was rather connected directly to the dendritic lumen (Figure 2C,D). A total of 53% of somatic spines were found to be stubby. In comparison, only 40% of dendritic spines were stubby spines. A large proportion of somatic (37%) and dendritic spines (40%) could be classified as mushroom spines that have a wide spine head on top of a much thinner spine neck connecting them to the cell lumen. This spine morphology is generally considered to be found in mature spine synapses (Pchitskaya and Bezprozvanny 2020). Finally, 11% of somatic and 20% of dendritic spines were considered long and filamentous. They had a prominent spine neck but a thinner spine head than mushroom spines. Examples of somatic spines from each group are shown in Figure 2E.

We next asked whether the morphological differences were accompanied by a difference in the spine metrics, measuring multiple characteristics (Figure 2F). Measurements indicated that the somatic spines were smaller than their dendritic counterparts in the septum (Figure 2G,H). Accordingly, they were on average 0.5‐µm long, with dendritic spines around 0.75 µm (Figure 2I). Despite the clear differences in measured spine area and length, the PSD width, membrane length, spine head, and spine neck width at the base of the spine did not differ between somatic and dendritic spines (Figure 2J–L). When comparing the number of SVs per presynaptic bouton opposed to the postsynaptic density of a spine, we found a similar number of SVs in boutons opposing somatic or dendritic spines (Figures 2M and S1I). The observed differences in spine morphology between somatic and dendritic compartments motivated us to examine whether they could be explained by changes in the stubby spine population. Notably, stubby spines located on the soma tended to be smaller than those found on dendrites. None of the other measurements were different between the two stubby spine types (Figure S1A–H).

### 3.3 Septal Spines Are Mainly Excitatory and Frequently Contain Organelles

**FIGURE 3:**
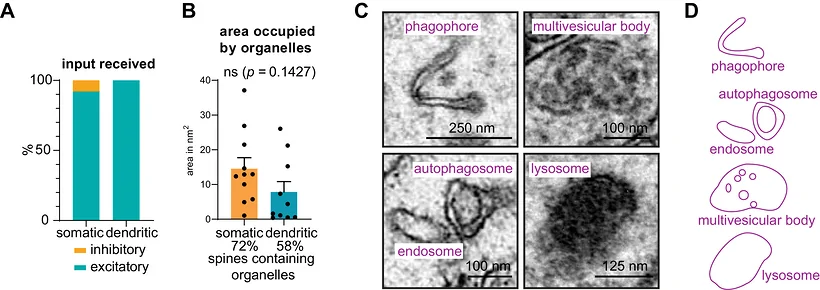
Septal spines are mainly excitatory and frequently contain organelles. (A) Share of spines receiving inhibitory or excitatory input as evidenced by a similarly electron‐dense PSD and presynaptic side for inhibitory synapses and an asymmetrically stronger signal in the PSD for excitatory synapses (*n* = 14 somatic and *n* = 22 dendritic spines; shown: cell averages, *N* = 2 animals). (B) Percentage of somatic or dendritic spines containing organelles and area occupied by organelles in spines containing organelles. No normal distribution according to the D'Agostino and Pearson test; Mann–Whitney *U* test: *p* = 0.1427. (C) Additional examples of organelles found in somatic spines. (D) Schematic representations of organelles seen in (C).

While dendritic spines are almost exclusively excitatory (McKinney 2010), they can occasionally contain synapses at the spine neck (Brusco et al. 2014), and perisomatic synapses are often inhibitory (Eyre et al. 2007; Szabó et al. 2010). We next characterized the spines as excitatory or inhibitory depending on the symmetry of the PSD and the opposing presynaptic site. A similarly electron‐dense presynapse and PSD were classified as an inhibitory synapse and a clearly more electron‐dense PSD as excitatory, as published previously (Colonnier 1968; E. G. Gray 1959; Gray 1969; Peters, Alan and Palay, Sandford L 1996; Peters and Kaiserman‐Abramof 1970; PING 2008). SVs were found at the presynaptic sites of all classified PSDs, excluding the possibility of having misclassified gap junctions or immature synapses as inhibitory ones. We found that the vast majority (90%) of somatic spines and all dendritic spines were excitatory in nature (Figure 3A) despite the predominance of inhibitory neurons within the septum.

Spine synapse plasticity depends on the availability of various membranous organelles that are generally considered to be shared between dendritic spines that are found in close proximity to each other (Cooney et al. 2002). It is unclear how the proximity of somatic spines to the organelle‐dense soma would affect the transport and subsequently the availability of organelles, which could affect their plasticity.

We found that 72% of all somatic spines contained organelles such as phagophores, multivesicular bodies, endosomes, autophagosomes, and lysosomes (Figure 3B–D). We did not find any example of a somatic spine containing a spine apparatus, an ER‐derived organelle typically found in stable spine synapses (Segal et al. 2010). A total of 58% of dendritic spines were also frequently found to contain various organelles (Figure 3B), but contained a rather distinct inventory from somatic spines. A list of organelles found in somatic and dendritic spine synapses is provided (Table 1). Spines could contain more than one organelle type at the same time, bringing up the sum of the percentages to more than 100%. Additionally, we found that the somatic spine area occupied by organelles was increased by stubby spines (Figure S2A).

**TABLE 1:** Percentage of somatic or dendritic spines positive for selected organelles. Note that the sum of organelles found can exceed 100% as some spines contained more than one organelle.

	Somatic spines	Dendritic spines
MVB	36%	0%
Endosome or lysosome	45%	0%
Autophagosomal	18%	0%
Ribosomes	9%	55%
Spine apparatus	0%	18%
Other	9%	36%

### 3.4 Development of Somatic Spines Occurs Independently of Extra‐Septal Input in Dissociated Septal Cultures

**FIGURE 4:**
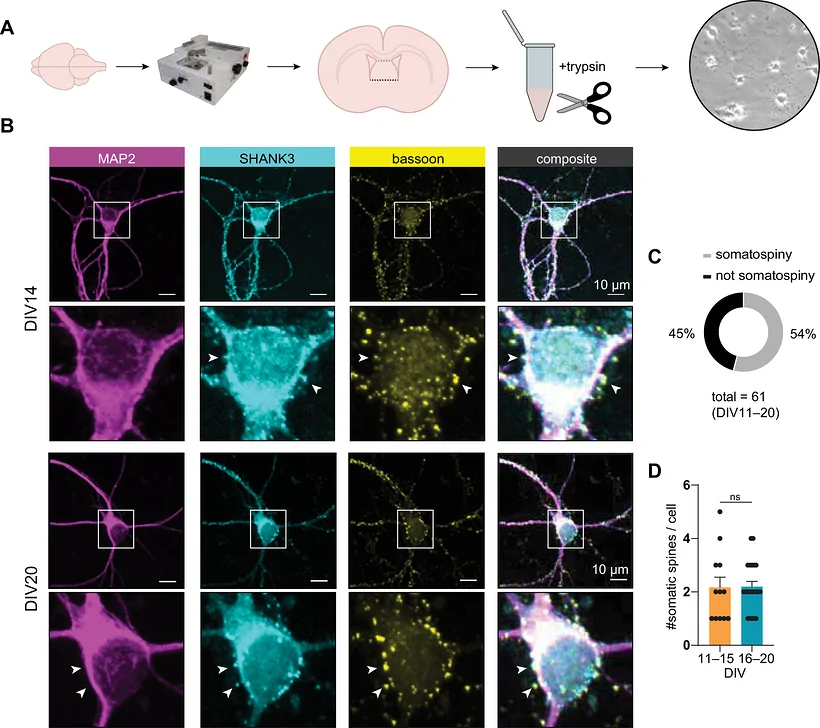
Somatospiny neurons develop independently of specific input in dissociated primary cultures. (A) Schematic showing the dissection method used to prepare septal dissociated cultures. (B) Dissociated septal neurons fixed at selected DIV, immunostained against the neuronal, somatodendritic marker MAP2 (magenta), postsynaptic scaffolding protein SHANK3 (cyan), and presynaptic marker bassoon (yellow). Scale bar, 10 µm. Indicated zoom‐ins of somata below the image. Somatic spines are indicated by arrows. (C) Percentage of neurons in septal dissociated culture stained as in B that bear somatospines (somatospiny) or not. *n* = 61 cells (*N* = 9 independent preparations). (D) Number of somatic spines at different stages in development found on somatospiny neurons as in B. Mann–Whitney *U* test: ns, DIV11‐15 versus DIV16‐20 *p* = 0.6822.

Although it has been suggested that septal somatic spines receive hippocampal input (Jakab and Leranth 1990a), it is presently unclear whether it is required for the development of somatic spines or whether it is an intrinsic feature and the somatospiny neurons are able to form somatic spines in the absence of hippocampal input. To investigate this question, we next set out to establish methods to be used to visualize somatic spines in neurons of the lateral septum that would allow us to access them and characterize their content. One suitable and easily observable model system for this is dissociated primary cultures, which are commonly used for studying hippocampal and cortical cells (Meberg and Miller 2003; Sahu et al. 2019). There are only a few, mainly early reports describing the preparation of dissociated septal cultures, and most focus on glial cells (Djemil et al. 2021; Dunnetf et al. 1986; Hartikka and Hefti 1988; Kenigsberg and Mazzoni 1995; Mazzoni and Kenigsberg 1991, 1994, 1996a, 1996b). In order to set up this model and ensure proper synaptic maturation of neurons, we optimized the protocol for culturing P0 and P1 rat septal cells (Figure 4A,B; see methods for details). In the initial preparation, a large number of cells did not adhere to the coated coverslips; we removed these cells with a washing step to remove debris (for more details, check Section 2). The cells could then be cultured until DIV15. In our cultures, we found that already at the young age of DIV7–8, around 58% of neurons were positive for the marker calbindin (Figure S3A,B), which is considered to be enriched in the septum (Kiss et al. 1997). At the same age, more than 60% of neurons were positive for GAD65. Both markers are known to be expressed in inhibitory neurons. Interestingly, not all calbindin‐positive cells were also positive for GAD65, making more than 60% of cells clearly of septal origin.

Here, we could combine genetic manipulation with subsequent immunostaining to understand the molecular composition of these somatic spines during development.

On average, we found that 54% (*n* = 33 somatospiny neurons of 61 total cells, *N* = 9 independent preparations) of septal neurons labeled with MAP2 and synaptic markers SHANK3 and bassoon were somatospiny neurons. This conclusion was supported by the presence of synaptic markers outside the bounds of the somatodendritic marker MAP2‐signal (Figure 4C). On average, we observed only a few somatic spines on somatospiny neurons with no significant differences between DIV11 and DIV20 (Figure 4D).

This suggested the ability of somatospiny neurons to develop somatic spines in the absence of extra‐septal, for example, hippocampal input.

### 3.5 Somatic Spines Observed in Organotypic Septal Slices Contain Classical Postsynaptic Markers

**FIGURE 5:**
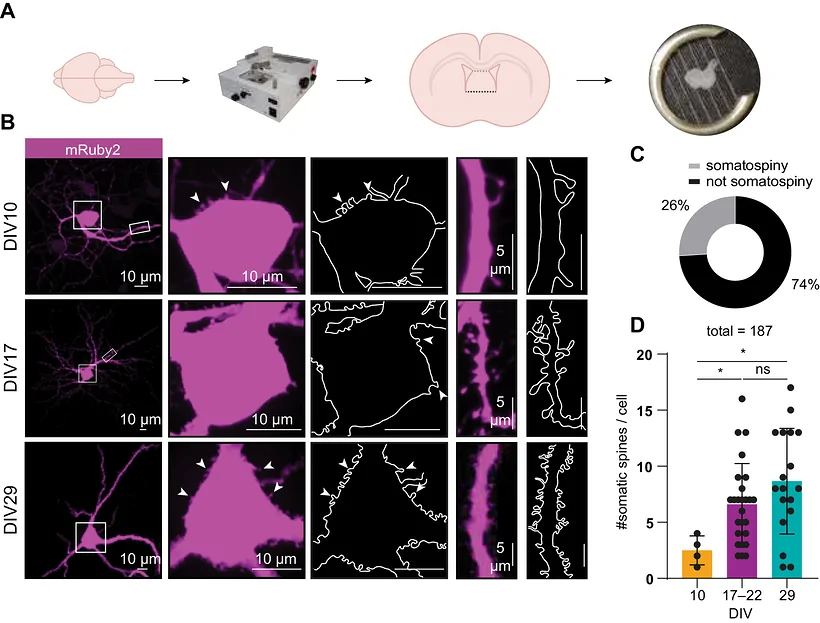
Somatic spines develop in organotypic septal slice cultures. (A) Schematic showing the dissection method used to prepare septal organotypic cultures. (B) Maximum projections of electroporated septal neurons expressing the cell‐fill mRuby2 and fixed at selected DIVs. Scale bar, 10 µm for overview and somatic images and 5 µm for dendrites. Outlines drawn in panels to the right of cell‐fill images. (C) Percentage of somatospiny neurons in septal organotypic culture and those without somatic spines. *n* = 187 cells (*N* = 15 animals). (D) Number of somatic spines at different stages in development found on somatospiny neurons as in (B). Mann–Whitney *U* test: *, DIV10 versus DIV17‐22 *p* = 0.0142; ns, DIV17‐22 versus DIV29 *p* = 0.1039; *, DIV10 versus DIV29 *p* = 0.0187.

While dissociated cultures are especially accessible, the topology, microenvironment, and connectivity patterns between cells are lost. To keep a similar level of accessibility, but simultaneously increase the level of complexity and move closer to the native connectivity, we next aimed to establish organotypic septal slices from postnatal rats (P4–7) (Figure 5A). To that end, we developed a protocol for their preparation, with the help of which we could maintain cells until DIV29. To identify a number of neurons, we sparsely labeled neurons using field electroporation of plasmid DNA encoding mRuby2, used as a volume marker (Figure 5B). We then quantified the number of somatic spines found on somatospiny neurons as well as the share of somatospiny neurons and compared our findings for different developmental stages (DIV10–29). We found that 26% (*n* = 48 somatospiny neurons of 187 total cells, *N* = 15 animals) of all randomly electroporated neurons were somatospiny neurons (Figure 5C). In contrast to dissociated cultures, we found an increase in the number of somatic spines on a somatospiny neuron during development (Figure 5D). At DIV10, only around 2 somatic spines could be found on a somatospiny neuron, increasing to ∼6 at DIV17–22 and to ∼9 at DIV29 (Figure 5D). Differences in cell density and connectivity between the model systems may account for the observed variations. On the other hand, these results suggest an ongoing spinogenesis in somatospiny neurons that does not require extra‐septal innervation.

**FIGURE 6:**
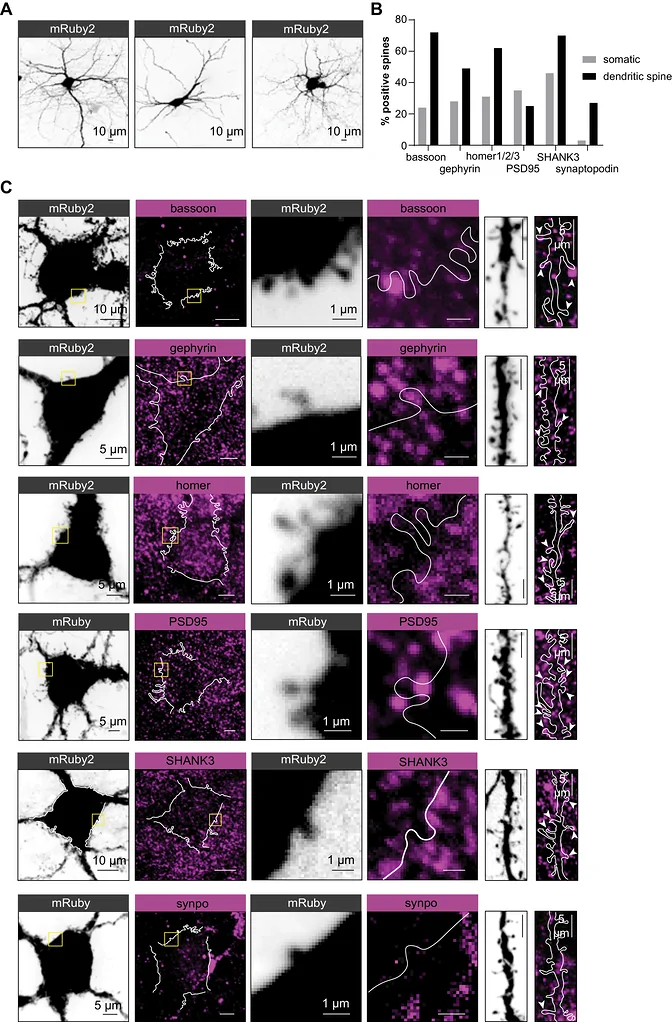
Molecular characterisation of somatic spines in organotypic septal slice cultures. (A) Maximum projections of electroporated septal neurons expressing the cell‐fill mRuby2. Scale bar, 10 µm. (B) Quantification of electroporated neurons as in (A) with somatic spines containing selected synaptic markers. (C) Electroporated septal slices fixed at DIV17‐29, immunostained for selected synaptic markers. Shown from left to right: somata, somata outlines and synaptic markers, somatic spine zoom‐ins, somatic spine outlines and synaptic markers, dendrites, and dendrite outlines and synaptic markers.

After confirming the validity of organotypic septal slices for the characterization of somatic spines, we sought to identify which synaptic markers are present in somatic spines. We thus stained for common synaptic proteins, including the presynaptic scaffolding protein bassoon, the inhibitory postsynaptic scaffolding protein gephyrin, and several excitatory postsynaptic proteins: Homer1/2/3, PSD95, and SHANK3, as well as the spine apparatus marker synaptopodin. Despite most somatic spines displaying mushroom or stubby spine‐like morphology and clearly containing PSDs (Figure 2), we found that many of them do not ubiquitously contain classical synaptic markers (Figure 6A–C). We found only around 24% of somatic spines that were positive for bassoon (Figure 6B,C). About 28% of spines were found to contain the inhibitory scaffolding protein gephyrin, 31% to contain Homer1/2/3, and 35% PSD95 (Figure 6B,C). Around 46% of somatic spines were positive for the excitatory scaffolding protein SHANK3, and only 3% contained synaptopodin. As a comparison, we also quantified the presence of our selected markers in dendritic spines of septal neurons (Figure 6B). Here, we found that 72% of spines were positive for the universal presynaptic marker bassoon. A total of 49% contained gephyrin, 62% Homer1/2/3, and only 25% PSD95. Seventy percent were positive for SHANK3, and only 27% contained synaptopodin. Altogether, this suggests that somatic spines exhibit greater variability in the presence of scaffolding proteins, which can be explained either by a potentially higher molecular diversity in this type of synapse or by the loss of extra‐septal presynaptic inputs over long‐term culturing.

## 4 Discussion

Here, we reaffirm the existence of septal somatic spines in the lateral septum. New approaches allowed us to identify the subcellular and molecular composition of septal somatic spines and to compare them to dendritic spines of the same region.

We first set out to classify whether all septal somatic spines were excitatory in nature. While dendritic spines are generally excitatory and known as the major excitatory input processor of the neurons (Yuste and Bonhoeffer 2004), somatic spines are less well characterized. Using TEM, we found evidence that both septal somatic and dendritic spines are excitatory when assessing the asymmetry of the PSD (Gray 1969; Colonnier 1968). In search of molecular markers for somatic spines, we established new protocols for the generation of dissociated cultures and organotypic slices, both of which are disconnected from extra‐septal networks following their dissection at P0/1 or P4–7, respectively. While such preparations result in a network that lacks input from other brain regions, these methods gave us the opportunity to study whether septal somatic spine formation existed as a built‐in program of the postsynaptic somatospiny septal neurons. We indeed found that somatic spines could develop without requiring input from any brain region other than the septum, especially in dissociated neurons, where they are reset to a stage preceding polarization and synaptogenesis. This indicated that the somatic spines developed independently of excitatory input, for example, from the hippocampus, and that the development of such a subcellular compartment is an intrinsic property of a subpopulation of septal neurons.

In vivo, somatic spines have been proposed to receive input from hippocampal somatostatin neurons as well as catecholaminergic neurons (Jakab and Leranth 1990a, 1991). This input could be replaced depending on available cells in in vitro systems, as in Agi et al. (2024), where it has been shown that axons self‐sort depending on available postsynaptic targets. To answer the question of whether in vivo septal somatic spines indeed receive synaptic input from the hippocampus, tracing experiments would be most suitable.

Additionally, the high density of synaptic markers in septal organotypic slices indicated a mature and healthy environment, but made it challenging to judge whether an individual somatic spine was positive for a specific marker. Additionally, we compared dendritic spines of the somatospiny cells to the somatic spines, placing the analyzed somatic and dendritic spines in close spatial proximity to each other. The vast majority of dendritic spines were positive for the universal presynaptic scaffolding protein bassoon and often other established postsynaptic synaptic markers. This indicated that the low share of somatic spines positive for the selected markers was not caused by methodological shortcomings, but reflected a decreased content of established synaptic proteins, possibly indicating a lesser degree of maturity of somatic spines in the absence of appropriate inputs. While we tested a number of different synaptic markers, we were not able to use more than two at the same time. Thus, our results could also indicate a high variability in the molecular composition of somatic spines. While we were able to see the PSD in EM, protrusions identified with confocal microscopy in organotypic slices that were negative for the selected markers could potentially also be non‐postsynaptic sites that might be more frequent than on dendrites (Arellano et al. 2007). The same, of course, also applies to Golgi stainings, which we used to count spine‐like protrusions (e.g., discernible spine neck and head). This would, however, likely exclude shaft synapses for which the spine head is similarly broad as the base of the spine neck. Golgi stainings, of course, present the advantage of better representing the in vivo organization.

A more complete overview of the molecular profile of somatospiny neurons could be achieved by patch‐sequencing, spatial transcriptomics, and single‐cell proteomics—but would be challenging to achieve due to the lack of a somatospiny neuron‐specific marker and the predominance of somatodendritic and axonal proteins in the resulting sample. Further analysis may reveal whether different scaffolding proteins may be part of the pre‐ or postsynaptic compartments of somatic spine synapses. Multiplexing analyses could be used to investigate whether somatic spines are a heterogeneous group of synapses that express different markers and thus only contain a select few of our observed markers.

We also found a developmental increase in somatic spine numbers in organotypic septal slices, but could not observe the same trend in dissociated cultures. This may be due to a higher cellular density, which often correlates with a higher density of synapses, indicating increased synaptogenesis or stabilization of the formed synapses. Additionally, differences in culturing media and initial age of animals (P1 for dissociated cultures, P4–7 for organotypic slices) at the time of preparation may contribute to differences between culture models. The timeline of somatic spine development in organotypic slices is comparable to, for example, hippocampal spine synapse development (Schünemann et al. 2025), and underlying mechanisms promoting spine development, such as cytoskeletal remodeling (Heinze et al. 2022; Matus 2000), are likely similar.

We were particularly interested in the occupancy of dendritic and somatic spines by different membranous organelles. Here, using electron microscopy, we are able to identify the organelle type through the detection of their shape. We found that somatic spines more frequently contained endosomes, lysosomes, organelles of the autophagosomal pathway, and multivesicular bodies (Table 1). The difference in organelles present pointed to a different underlying physiology and membrane turnover, associated with increased memory capacity (Frank et al. 2018). Surprisingly, we have not found a single example of a spine apparatus, usually indicating stable synapses, in somatic spines. Due to their positioning at the soma, somatic spines would be expected to have an especially large impact on the cellular output, as shown by the strong impact of, for example, axo‐axonic synapses at the axon‐initial segment on neuronal output (Nathanson et al. 2019).

The septal somatic spine organelle content stood in contrast with that of septal dendritic spines, which were more often positive for ribosomes, which we could only rarely (9%) find in septal somatic spines. Such differences indicate that local protein synthesis might play a role for septal dendritic spines, but for somatic spines, transport of proteins from the soma may be sufficient. To our surprise, we found that septal somatic spines were smaller than dendritic spines on neurons of the same region. This was in contrast to somatic spines containing organelles more often, while the individual area occupied by organelles was comparable between the different spine types.

Our TEM quantification was based on electron density of the postsynaptic density that exhibits a stronger signal at excitatory postsynapses, causing the synapse to appear asymmetric when comparing the pre‐ and postsynaptic site. To gain an additional level of certainty, immunogold labeling could be performed using antibodies raised against proteins typically found at the excitatory postsynapse, such as PSD95 and Homer, or those typically found at the inhibitory postsynapse, such as gephyrin.

Even prior to TEM, Golgi staining is arguably the oldest method of visualizing neurons in the brain (Golgi 1885) and can still serve as a method to do so reliably. This method is, however, prone to artifacts that are often visible as black dots of ∼1 µm diameter, similar to those of spine synapses. This already caused Camillo Golgi to hypothesize that dendritic spines are staining artifacts and not structures found on dendrites (Golgi 1885). While it is undisputed that dendritic spine synapses exist and are quite clearly visible on dendrites of Golgi‐stained samples, somatic spines are less easily distinguished from artifacts surrounding the neuronal somata. We could find evidence of somatic spines in Golgi‐stained sections from the septum of an adult rat; however, we did not quantify their occurrence since not every assumed somatic spine was undoubtedly a spine rather than a staining artifact. For the identification of spines, we specifically checked for the presence of a spine neck, which, in combination with a spine head, made it less likely to be an artifact. In combination with other methods, we can confidently say that the adult rat lateral septum contains a vast number of somatospiny neurons. For further investigation, we decided to focus on fluorescent microscopy and TEM to visualize single neurons and the composition of the somatic spines in multicolor images.

## 5 Conclusion

In this work, we confirm the existence of somatic spines in the lateral septum and characterize them in different model systems in which they can develop independently of input from brain regions other than the septal area. They are smaller than dendritic spines but more frequently contain membranous organelles. Despite being excitatory in nature, they are generally negative for most excitatory synaptic markers, but in dense networks that would mimic conditions in the brain, they show a similar developmental timeframe to dendritic spines. While the investigation of somatic spines has subsided in the last few years, the lateral septum is not the only region containing somatospiny neurons. Notably, they are found on ganglionic neurons in the peripheral nervous system in the hypothalamus, on Purkinje cells in the cerebellum, and in the adult hippocampus (Hirano et al. 1977; Ifft and McCarthy 1974; Shoop et al. 1999; Wenzel et al. 1994). The new approaches established here and molecular characterizations of somatic spines provide a comprehensive foundation to further elucidate the enigmatic somatic spines of septal neurons. It opens up the possibility of manipulating somatic spines and somatospiny neurons to investigate their role. It could connect a specific synapse type to a broader network, and their impact could be tested in behavioral analyses probing social memory.

## Conflicts of Interest

The authors declare no conflicts of interest.

## Data Availability

The data that support the findings of this study are available from the corresponding author upon reasonable request.
